# Comparison Between Flat and Round Peaches, Genomic Evidences of Heterozygosity Events

**DOI:** 10.3389/fpls.2019.00592

**Published:** 2019-05-14

**Authors:** Qiuping Tan, Xiao Liu, Hongru Gao, Wei Xiao, Xiude Chen, Xiling Fu, Ling Li, Dongmei Li, Dongsheng Gao

**Affiliations:** ^1^College of Horticulture Science and Engineering, Shandong Agricultural University, Tai’an, China; ^2^State Key Laboratory of Crop Biology, Shandong Agricultural University, Tai’an, China; ^3^Fruit Innovation of Modern Agricultural Industry Technology System in Shandong Province, Shandong Agricultural University, Tai’an, China; ^4^College of Horticulture, Nanjing Agricultural University, Nanjing, China

**Keywords:** *Prunus persica*, fruit shape, bud sport, genome-wide association study, next generation sequencing

## Abstract

Bud sports occur in many plant species, including fruit trees. Although they are correlated with genetic variance in somatic cells, the mechanisms responsible for bud sports are mostly unknown. In this study, a peach bud sport whose fruit shape was transformed to round from flat was identified by next generation sequencing (NGS), and we provide evidence that a long loss of heterozygosity (LOH) event may be responsible for this alteration in fruit shape. Moreover, compared to the reference genome, we identified 237,476 high quality single nucleotide polymorphisms (SNPs) in the wild-type and bud sport genomes. Using this SNP set, a long LOH event was identified at the distal end of scaffold Pp06 of the bud sport genome. Haplotypes from 155 additional peach accessions were phased, suggesting that the homozygous distal end of scaffold Pp06 of the bud sport was likely derived from only one haplotype of the wild-type flat peach. A genome-wide association study (GWAS) of 127 peach accessions was conducted to associate a SNP found at 26,924,482 bp of scaffold Pp06 to differences in fruit shape. All accessions with round-shaped fruit were found to have an A/A genotype, while those with A/T, or T/T genotypes had flat-shaped fruits. Finally, we also found that 236 peach accessions and 141 Prunus species with round-type fruit were found to have an A/A genotype at this SNP, while 22 flat peach accessions had an A/T genotype. Taken together, our results suggest that genes flanking this A/T polymorphism, and haplotyped carrying the T allele may determine flat fruit shape in this population. Furthermore, the LOH event resulting in the loss of the haplotype carrying the T allele may therefore be responsible for fruit shape alteration in wild-type flat peach.

## Introduction

Because of its small genome size and relatively short juvenile period, peach (*Prunus persica* L.) is an important model species in the Rosaceae (Shulaev et al., 2008). Peach fruit can be classified as flat or round, with the flat shape being dominant over the round shape (Lesley, 1940). Previous linkage maps revealed that fruit shape is controlled by a S-locus that co-segregates with molecular markers MA040a and MA014a at the distal end of scaffold Pp06 (Dirlewanger et al., 2006). It has also been found that this S-locus was associated highly with SSR marker UDP98-412 (Picañol et al., 2013). Moreover, it has recently been reported that fruit shape variance is strongly associated with an A/T polymorphism at a locus located at 25,060,196 bp of scaffold Pp06. In addition, fruits with a homozygous T/T genotype at this locus are round, whereas fruits with heterozygous (A/T) and homozygous A/A genotypes at this locus are flat (Cao et al., 2016). Fruit shape has also been related to a 10 kb deletion that affects the function of the leucine-rich receptor-like kinase (LRR-RLK) gene *Prupe.6G281100* (López-Girona et al., 2017). The ortholog of this gene in *Arabidopsis thaliana* is responsible for regulating meristem size and organization (Mandel et al., 2016).

Bud sports are a common somatic mutation found in many plant species, including fruit trees and provide many novel variants that can be bred by plant breeders. Fruit shape differences occasionally result from bud sports (López-Girona et al., 2017). Although previous studies have suggested that bud sports may occur both on the whole genome and individual gene levels (Foster and Aranzana, 2018) to date the precise molecular mechanisms responsible for bud sports that result in fruit-shape alteration are unknown.

Loss of heterozygosity (LOH) events are common genetic events found in many types of cancer (Koufos et al., 1985; Yokota et al., 1987; Deng et al., 1996; Gatto et al., 2014; Muzumdar et al., 2016). Early studies that used microsatellite-based whole-genome analyses to study LOH events in cancer found that cancer progression often resulted from LOH events at particular loci (Koufos et al., 1985; Naylor et al., 1987; Emi et al., 1992). Later studies used SNP arrays to analyze LOH event prevalence in cancer cells, and found that extensive LOH occurrence was indicative of instability of the whole genome rather at individual loci (Lindblad-Toh et al., 2000). In contrast to cancer biology, few LOH events have been documented in plants. Wang et al. (1999) reported an LOH event that occurred at a specific locus in F1 rice hybrid plants, and Xu et al. (2007) used whole genome data for rice hybrids to identify LOH events in F1 progeny. A third report studied LOH events in *A. thaliana* regenerants, and found that LOH events had occurred at individual loci distributed across three of five scaffolds of the *A. thaliana* genome (Zimina et al., 2016). LOH events have also been documented at the berry color locus in grapevine (Vezzulli et al., 2012; Pelsy et al., 2015; Carbonell-Bejerano et al., 2017; Migliaro et al., 2017).

Since markers can provide information about the sequences flanking LOH loci, the availability of new genetic markers can help to more efficiently explore LOH events in plants. NGS methods, which can provide hundreds of millions of informative SNP markers for genetic studies (Metzker, 2010), have been used to study peach domestication and to characterize the genetic differences between peach varieties (Verde et al., 2013; Cao et al., 2014). In addition, GWAS analyses are now commonly used to examine the genetic variance responsible for important agronomic traits in *A. thaliana* (Atwell et al., 2010), rice (Huang et al., 2010), and peach (Micheletti et al., 2015; Cao et al., 2016).

In this study, we used an NGS-focused approach to study genomic differences between flat peach and its bud sport genome. We obtained an SNP set that we used to explore the genomic mechanism responsible for fruit shape variance in the bud sport. In addition, a GWAS was performed to relate genetic variance to fruit shape variance in cultivated peach. Finally, we discuss the possible mechanisms that may be responsible for the LOH event that we identify as determining fruit shape.

## Materials and Methods

### Plant Materials

A single 8-year-old specimen of the flat peach cultivar “Zhaoyue” (*P. persica* L.) was grown in the orchard of the experimental station of Shandong Agriculture University, Tai’an, China. The base of one branch of this tree produces normal flat-shaped fruit while the top of the same branch bears round-shaped fruit (Supplementary Figure S1). The focal tree was maintained as other peach trees in the orchard were. The bud sport was stable for at least 3 years.

### Sample Collection and DNA Extraction

**FIGURE 1:**
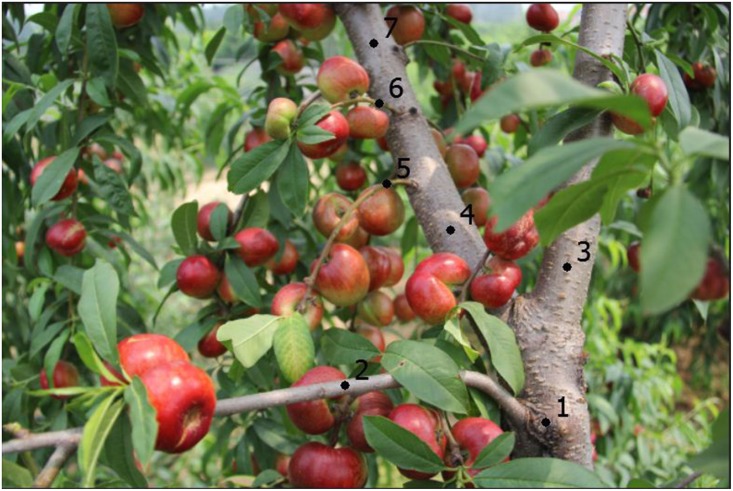
The phenotypic characterization of the flat peach and its bud sport at maturity. Label 1 indicates the main branch, under this label all fruits produced were flat; labels 2, 3 show two lateral branches containing flat fruits; label 4 indicates a bud sport branch, whose two sub-branches (labels 5, 6) bore both flat and round fruits; branches above label 7 bore round-type fruit only. This picture was photographed at 66 DAB (days after blooming) at fruit ripening.

To avoiding sampling chimeras, we selected only fruits produced at locations far from the intermediary shoots that contained both round and flat peaches. Thus we obtained a bud sport-type sample by selecting round-type fruit from the topmost branches (indicated by label 7 of Figure 1), while wild-type flat fruits were sampled from the bottommost branches (indicated by label 1 of Figure 1). More than 50 healthy fruits were collected from both wild-type and bud sport mutant shoots approximately 90 days after flowering, which is when fruit shape is definitively established. Fruit mesocarps from five or more fruits were pooled for both the wild-type and bud sport mutant samples. Mesocarps not leaves were sampled since the mesocarp develops only from LII layer cells. At least 10 biological duplicates were obtained for both samples. All samples were frozen in liquid nitrogen and stored at -80°C for later DNA extraction. DNA was extracted from each sample using a Tiangen DNA extraction kit, as per the manufacturer’s instructions. Next, 10+ DNA samples were pooled for NGS sequencing. Two samples were prepared: one included DNA pooled from wild-type fruits while the other included DNA pooled from bud sport fruits.

### NGS Sequencing

Paired-end libraries were prepared for each sample according to the standard Illumina procedure. 125 cycles per read were sequenced using a HiSeq2000 system. All raw reads were mapped against the peach reference genome v2.0 (Verde et al., 2013) using BWA-MEM v0.7.12 (Li, 2013). Only reads with >30 mapping quality were included for further analysis. Mapped reads were sorted by SAMtools v1.2 (Li et al., 2009) and duplicates were removed with Picard v1.2^1^. SNP calling was performed using GATK v3.4 (McKenna et al., 2010). Reads from the reference genome were also included in our SNP calling pipeline to estimate the robustness of our procedure.

After calling using the GATK pipeline, a series of filter steps was implemented to retain as many true SNPs and remove as many false SNPs as possible. First, at the sample level, a genotype quality threshold of 20 was applied to raw variance sets to exclude low quality genotypes. Second, any SNPs with missing values were also excluded. Third, at the variance level, a variety of parameters were applied to raw datasets to compare the filtered and unfiltered variances. Parameter values were adjusted to retain true SNPs and exclude false SNPs. The final filter thread was:

QD < 10.0||MQ < 59.9||MQ > 60.1||FS > 15.0||MQRankSum < -0.04||MQRankSum > 0.04||ReadPosRankSum < -1.5||SOR > 2.0||ClippingRankSum < -0.3||ClippingRankSum > 0.3.

This stringent multiple-step filtering approach guaranteed that we obtained a higher calling rate of true SNPs.

### LOH Detection

To conveniently identify differences between two genomes sourced from two samples, SNPs marker genotypes were coded numerically. Using a scheme where 0 represents a SNP that is homozygous for the reference allele, 1 represents a SNP that is heterozygous for the reference and variant alleles, and 2 represents a SNP that is homozygous for the variant allele. We then examined the distribution of numerical codes throughout the genome. An LOH event was deemed to have occurred when the SNP genotype of the wild-type was 1 but 0 or 2 in the bud sport. Since some SNP genotypes may represent false negatives resulting from the sequencing or calling pipeline, we required LOH events to extend longer than 5 continuous SNPs. LOHs events were identified using the vcftools software package (Danecek et al., 2011; Supplementary Table S1). We also called small indels and large structural variants to validate the LOH event occurring (Supplementary Methods).

### Comparisons of Genotype Depth in the Two Samples

To conveniently compare differences in genotype depth between the two samples, we calculated the ratio of the genotype depth of the bud sport mutant to that of the wild type. The distribution patterns of this ratio at both the whole genome and the local scaffold Pp06 levels were analyzed further.

### Haplotype Construction Using the Whole-Genome SNP Set

Sequencing reads from 222 SRA runs of peach accessions were downloaded from the European Bioinformatics Institute database^2^. Additional sequencing reads from 23 SRA runs of peach accessions with low sequencing depth were obtained from GDR^3^, and paired reads from wild flat peach and the bud sport mutant reported here were also included. The raw reads from 247 SRA runs (Supplementary Table S2) were individually mapped against the peach genome 2.0 using novoalign software^4^ with the following parameters:

Novoalign-d Prunus_persica_v2.0.a1_scaffolds-t 15, 3-H 20 - softclip 20-r Random - hlimit 8-p 5, 20 - matchreward 3-k-aGATCGGAAGAGCGGTTCAGCAGGAATGCCGAG; ACACTCTTTCCCTACACGACGCTCTTCCGATCT

After mapping, bam files were individually sorted and duplicates were removed. Accessions with the same sample identity were integrated into the same accession, resulting in 157 unique accessions. The raw variance of the 157 peach accessions (Supplementary Table S3) was called using GATK tools v3.4 (McKenna et al., 2010). The SNP set was extracted for downstream analyses. The set of 230,102 SNPs common to the largest 8 scaffolds found in both this approach and in the above pipeline [determined using BWA-MEM (Li, 2013)] was extracted using vcftools (Danecek et al., 2011). Haplotype construction using the resulting SNP set and genomic data from the 157 accessions was performed using BEAGLE software version 4.1 (Browning and Browning, 2007), using 100 iterations with a sliding window of 10,000 SNPs. Next, phased haplotypes of wild type flat peach and the bud sport mutant were extracted to further explore the genetic variance of the bud sport mutant relative to the wild type. This variance was visualized using Circos v0.69 (Krzywinski et al., 2009). 7 pair of primers throughout the distal end of scaffold Pp06 were designed to validate the origin of haplotype of bud sport (Supplementary Methods, Supplementary Table S7).

### SNP Calling for GWAS

Based on a preliminary PCA analysis, 10 wild accessions, 18 low-depth accessions (including 5 accessions that were integrated), and two accessions with distinct divergence were all excluded prior to conducting a GWAS. The resulting 217 SRA runs were individually mapped against the peach genome version 2.0 (Verde et al., 2013) using BWA-MEM (Li, 2013) with the parameters (-M -t 4). Only reads with MQ scores ≥60 were retained and converted to bam files. After converting, sorting, reduplicate-removing, and header-reshaping were successively conducted on each bam file, the files were then integrated according to sample identity. This resulted in a set of 127 bam files (Supplementary Table S4). We used Varscan (Koboldt et al., 2012) to determine the SNP variance of the resulting 127 accessions. The parameters used were as follows:

samtoolsmpileup-B-q1|java-jarvarscanmpileup2snp -min-coverage2 - min-reads21 - min-avg-qual15 - min-var-freq0.25 - min-freq-for-hom0.75 - p-value0.99 - output-vcf 1. The SNP set was then extracted for downstream analysis.

### PCA of 127 Peach Cultivars

The population structure of the 127 peach cultivars was estimated by principal component analysis (PCA) implemented by EIGENSTRAT v 6.1 (Price et al., 2006) on an LD-pruned pseudomolecule SNP set that included 21,728 SNPs. The top ten principal components were used for downstream genome-wide association mapping. The LD between SNPs in the 127 cultivars was evaluated using squared Pearson correlation coefficients (r^2^) as calculated by the -r2 command of PLINK v1.9 (Purcell et al., 2007). An LD heatmap surrounding GWAS peaks was constructed using the *LDheatmap* R package (Shin et al., 2006).

### GWAS of 127 Peach Cultivars

The full pseudomolecule SNP set (6,138,928 SNPs) was filtered to retain SNPs with call rates >75% and minor allele frequencies >5%, resulting in 431,028 SNPs. This set was used to conduct the GWAS. Mixed-model association analysis was conducted in EMMAX (Zhou and Stephens, 2012) using a Balding-Nichols kinship matrix. Bonferroni-adjusted *P*-values (with an overall significance threshold of 0.05) were used for significance testing. The top ten EIGENSTRAT principal components were used as covariates. Manhattan and quantile–quantile plots were generated by the *qqman* R package (Turner, 2014).

### SNP Phasing in Additional Peach Accessions and Prunus Species

We designed primer pairs to amplify the genomic interval flanking the SNP found at 26,924,482 bp of scaffold Pp06 to phase this SNP site with sequence data from 258 additional peach accessions (Supplementary Methods, Supplementary Table S5). In addition, a 101 bp sequence centered on this SNP was obtained from the sixth intron of *Prupe.6G292200* of the Lovell reference genome v2.0 (Verde et al., 2013). This sequence was then used to BLAST against the NCBI SRA dataset containing sequence data from all 141 available Prunus species (Supplementary Table S6).

### Availability of Data and Materials

The dataset supporting the conclusions of this article is available in the sequence read archive (SRA) database^5^.

## Results

### Phenotypic Characterization of the Flat Peach and Its Bud Sport

A single flat peach with round-type fruits was observed in our fruit orchard (Tai’an, China). The base of this tree (below Label 1) was bearing just flat-type fruits (Figure 1, Label 1). Moreover, two lateral branches also bore flat-type fruit (Figure 1, Labels 2, 3). The main branch (i.e., Labels 4, 7) was a fruit-shape alteration bud sport. Two lateral branches on the bud sport branch showed both flat- and round-type fruits (Figure 1, Labels 5, 6). The higher branches (i.e., those found above Label 7) on the bud sport branch showed only round-type fruits (Figure 1, Label 7).

### The SNP Calling Pipeline Was Highly Robust

We sequenced the whole genomes of the wild-type and bud sport peaches, generating ∼12 Gb of raw data for each sample. After mapping the reads to the peach reference with mapping quality filter ≥60, we obtained an average coverage depth of ∼28× and ∼26× for the wild-type and bud sport mutant genomes, respectively. More than 85% of the genome sequence was covered by at least 4 reads for each sample. Calling SNPs from short reads remained challenging, and we therefore included reads from the double haploid Lovell reference genotype (SRR502985) to control our SNP calling pipeline. A total of 237,476 high quality SNPs were obtained after quality control procedures were performed. As shown in Table 1, after mapping double haploid reads against the reference genome, we observed 455 SNP inconsistencies, yielding a putative calling inconsistency rate of 2.02E-6. However, some of these inconsistencies may be due to errors in the reference assembly. We consider this to be the case for most of the 397 homozygous SNPs found in the double haploid genome. The other 58 SNPs were heterozygous and appear to be aligned to paralogous sequences. Thus, the adjusted rate of false positive SNPs was 2.57E-7.

**Table 1:** SNP discordance between reads from the double haploid and reference genomes.

State	Discordance	Called SNP	Size of genome	Discordance rate	SNP density
Het^a^	58	237,476	225,694,811	2.57E-7	1.05E-3
Hom^b^	397	237,476	225,694,811	1.76E-6	1.05E-3
Total	455	237,476	225,694,811	2.02E-6	1.05E-3


*^*a*^Represents heterozygous SNPs.*

*^*b*^Represents homozygous SNPs.*

As shown in Table 2, the observed rate of inconsistency between the bud sport and wild-type genotypes was very low, suggesting that our SNP calling pipeline was highly robust. The numbers of genotypic inconsistencies differed among scaffolds, and the total inconsistency was approximately 4.52E-7 when we excluded data from the distal end of scaffold Pp06. This value is similar to that of reads from the double haploid reference genotype compared with the reference genome (2.57E-7, Table 1). As shown in Table 2, SNPs were unevenly distributed throughout the genome (1 SNP/950 bp on average); we found that scaffold Pp02 carried the most SNPs (1 SNP/480 bp on average), and scaffold Pp08 had the fewest SNP (1 SNP/3.2 kb on average). Furthermore, effective prediction of gene variants identified 9,056 non-synonymous SNPs, 8,185 synonymous SNPs, and 243 SNPs with high effect (i.e., those with premature stop codons, changed translation start sites, or changed splicing positions).

**Table 2:** Genotype discordance between wild-type and bud sport genomes.

Scaffold	Discordance	Called SNP	Size of scaffold	Discordance rate	SNP density
Pp01	7	35,297	47,851,208	1.46E-7	7.38E-4
Pp02	54	63,329	30,405,870	1.78E-6	2.08E-3
Pp03	6	23,281	27,368,013	2.19E-7	8.51E-4
Pp04	10	21,835	25,843,236	3.87E-7	8.45E-4
Pp05	4	24,476	18,496,696	2.16E-7	1.32E-3
Pp06^a^	14	35,486	30,767,194	4.55E-7	1.15E-3
Pp07	7	26,451	22,388,614	3.13E-7	1.18E-3
Pp08	0	7,101	22,573,980	0	3.15E-4
Remaining	0	220	NA^b^	0	NA^c^
Total	102	237,476	225,694,811	4.52E-7	1.05E-3


*^a^Excluded data from the distal end of scaffold Pp06 (from ∼22,202,387 to the telomere).*

*^b,c^Indicate data that was not collected.*

### A Long LOH Event Was Identified at the Distal End of Scaffold Pp06

**FIGURE 2:**
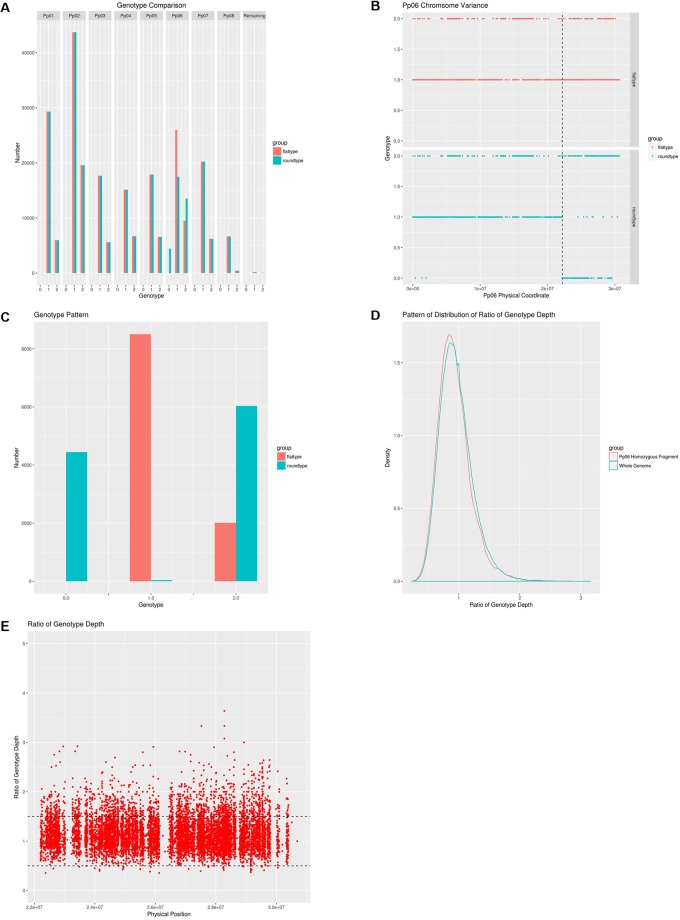
A comprehensive analysis of the genetic differences between the wild-type and the bud sport mutant. **(A)** Whole genome pattern comparison between the wild-type and the bud sport mutant. Wild-type (flat fruit shape) is shown in red, bud sport mutant (round fruit shape) is shown in blue. Homozygous reference is numbered as 0 and homozygous variance is numbered as 2, heterozygote is numbered as 1; **(B)** Genotype pattern comparison between the wild-type and the bud sport for scaffold Pp06. Wild-type is shown in red and the bud sport mutant is shown in blue. The transition site is located at 22,195,188 bp; **(C)** Genotype pattern comparison between the wild-type and the bud sport at the distal end of the Pp06 chromosome. Wild-type is shown in red and the bud sport mutant is shown in blue; **(D)** Pattern of distribution of ratio of genotype depth between bud sport and wild-type at both local and whole genome level. The distribution pattern of the genotype depth ratio between the bud sport and the wild-type on the local level is shown in red and the distribution pattern of the genotype depth ratio between the bud sport and the wild-type on the whole genome level is shown in blue. The local segment is the distal end of scaffold Pp06 (from 22,195,188 bp to the telomere). The whole genome level includes all chromosomes; **(E)** The genotype depth ratio between the bud sport and the wild-type on the distal end of the Pp06 chromosome.

Despite the fact that the number of inconsistencies between the bud sport and wild-type genotypes was very low for all scaffolds except Pp06, Pp06 itself attracted our curiosity by showing many more inconsistencies (Figure 2A). Compared to the wild-type, scaffold Pp06 of the bud sport mutant showed a reduction in the number of heterozygous SNPs and an increase in the number of homozygous SNPs (Figure 2A). Using our pipeline, we found 35,486 SNPs in scaffold Pp06, of which 8,501 showed discrepancies at thedistal end of scaffold Pp06 between the bud sport and wild-type genotypes (Table 3). This was three orders of magnitude higher than that of the other parts of the genome.

**Table 3:** Genotype discordance between wild-type and bud sport on scaffold Pp06.

Scaffold	Discordance	Called SNP	Size of scaffold	Discordance rate	SNP density
Proximal end of Pp06	14	24,971	22,202,387	6.31E-7	1.12E-3
Distal end of Pp06	8,501	10,515	8,564,807	9.93E-4	1.23E-3
Whole Pp06	8,515	35,486	30,767,194	2.77E-4	1.15E-3


As shown in Table 3, after comparing the genotype consistency of the wild-type, and bud sport mutant on the whole-genome scale, we found that genotype consistency was very high when we excluded the distal end of scaffold Pp06 (i.e., from ∼22,202,387 bp to the telomere). At the distal end of scaffold Pp06, we found a total of 10,515 SNPs. Of these, 8,501 were heterozygous in the wild-type, 8,465 were homozygous in the bud sport mutant, and the other 36 SNPs were heterozygous in the sport mutant. These 36 SNPs may be false negatives, generated by faults intrinsic to DNA sequencing or to the SNP calling pipeline, which therefore would give a rate of 4.19E-6 (∼8.6 M). The remaining 2,014 SNPs were homozygous in both the wild-type and the bud sport mutant. Thus, at least 99.66% of all SNPs (10,479) were not against the conclusion that the distal end of scaffold Pp06 shows signs of a LOH event. A LOH event occurring at the distal end of scaffold Pp06 of bud sport was further validated using Indel and DEL structural variants (Supplementary Figures S2–S5).

### Genotypes Were Significantly Different at the Distal End of Scaffold Pp06

We plotted a genotype distribution pattern along scaffold Pp06 and found that for the sequence running from 0∼22,202,387 bp of scaffold Pp06, there were no significant differences between the wild-type and bud sport mutant genotypes (Figure 2B). However, the remaining ∼8.6 Mbp sequence at the distal end showed significant differences between these genotypes. Within this interval, the sequence is mostly heterozygous in the wild-type and homozygous in the bud sport mutant.

Furthermore, a dataset consisting of 10,515 SNPs showed that the genotype at the distal end of scaffold Pp06 was significantly different between the wild-type and the bud sport mutant (Table 3). In the wild-type, there were only two genotypes: heterozygous SNPs (expressed as 1), and variant homozygous SNPs (expressed as 2); of the SNP loci tested, 8,501 SNPs were found to be heterozygous, and 2,014 SNPs were variant homozygous. However, in the bud sport mutant, the number of variant homozygous SNPs increased by ∼4,000 and the number of reference homozygous SNP increased by >4,400. Most of these differences were not present in the wild type.

### Genotype Depth of the Mutant Was Essentially the Same as That of the Wild-Type

Because deletions can cause heterozygous to homozygous conversions of runs of DNA, we plotted the genotype depth ratio (wild-type/bud sport) between the homozygous distal end of scaffold Pp06 and whole genome (Figure 2D). We found a similar distribution pattern with a peak at about 0.8, although we found more SNPs with ratios lower than 0.8 and fewer SNPs with a ratio of greater than 0.8 on the homozygous distal end of scaffold Pp06.

We further checked the genotype depth at the distal end of scaffold Pp06 between the wild-type and bud sport mutant genotypes (Figure 2E). We calculated the depth ratio (wild-type/bud sport) between these two samples and found that most SNPs centered around 1. More than 95% of all SNPs (9,991 in total) showed ratios between 0.5 and 1.5 and more than 63% of all SNPs (6,636 in total) were less than 1. This analysis showed that the genotype depth of the bud sport mutant was essentially the same as that of the wild-type. From this we speculated that it was unlikely that the homozygous scaffold was caused by deletion.

### Haplotype Analysis of 157 Peach Accessions

**FIGURE 3:**
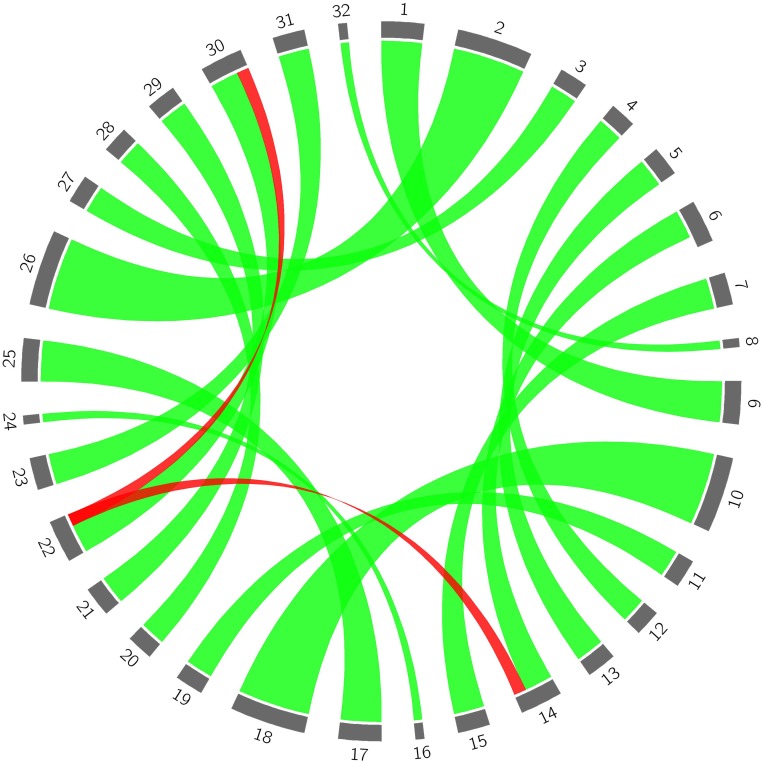
The origin of genetic variance of the bud sport mutant compared to wild flat peach. 1–8 represent haplotypes of each chromosome in the wild flat peach genome, respectively; 9–16 represent haplotypes of each chromosome in the bud sport genome; 17–24 represent haplotypes of each chromosome in wild flat peach; 25–32 represent the haplotypes of each chromosome in the bud sport mutant, respectively. Green lines represent normal genetic transfer from wild flat peach to the bud sport mutant and red lines represent the relationship between the distal end of scaffold Pp06 between the wild-type and bud sport mutant.

With the exception of the distal end of scaffold Pp06, all parts of the 8 largest scaffolds of the bud sport mutant genome were derived from the corresponding parts of the wild flat peach genome (Figure 3). With respect to the distal end of scaffold Pp06, we found that both of the segments of the bud sport mutant (i.e., red ribbons 14 and 30) were derived from the same segment of wild flat peach (i.e., red ribbon 22). Moreover, another haplotype of the distal end of scaffold Pp06 (i.e., number 6) in wild-type flat peach was absent in the bud sport genome. These findings suggest that the two (identical) haplotypes of the distal end of scaffold Pp06 of the bud sport genome were derived from only one haplotype of the corresponding chromosomal segment of wild flat peach. The other haplotype (i.e., number 6) was not transferred to the bud sport genome (Supplementary Data Sheet S2).

### PCA of 127 Peach Accessions

**FIGURE 4:**
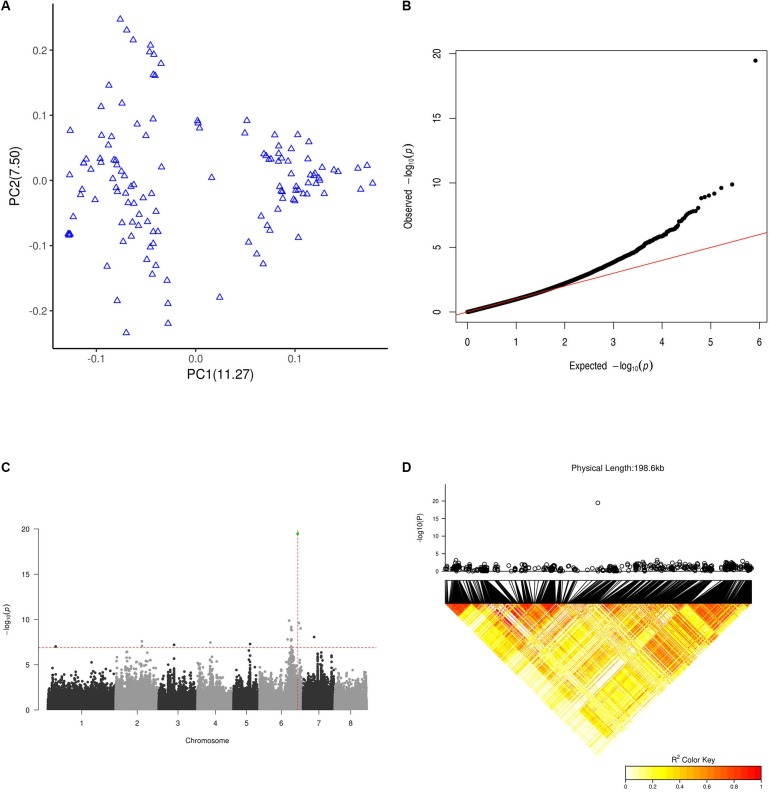
GWAS results for flat fruit shape. **(A)** PCA plot for the 127 GWAS peach cultivars based on a whole-genome LD-reduced SNP set. PC1 and PC2 indicate the scores of principal components 1 and 2, respectively. Values in parentheses indicate the percentage of variance in the data explained by each principal component. **(B)** QQ plot of the GWAS on the flat fruit shape trait. **(C)** Manhattan plot of GWAS results for flat fruit shape. The *X* axis represents the longest 8 scaffolds of the peach genome. The horizontal dashed line is the threshold value after abbirdx-adjustment (*P* = 7.2). The vertical dashed red line shows the location where the association signal was the most significant (*P* = 19.8). The green dot shows the SNP at 26,924,482 of scaffold Pp06 with the most significant signal. **(D)** LD pattern of SNPs within a 200 k flanking interval of the SNP at position 26,924,482 of scaffold Pp06. The top panel shows a local 200 k Manhattan plot for peach fruit shape surrounding the SNP at position 26,924,482 of scaffold Pp06. The bottom panel shows the LD pattern of SNPs within a 200 k interval surrounding the SNP at position 26,924,482 of scaffold Pp06.

Using the LD-reduced set of SNPs from the whole genome, we performed PCA to quantify the population structure of all 127 peach accessions. The principal component score plot showed a continuous distribution without any distinct clusters (Figure 4A), indicating that the accessions we examined did not represent a highly structured population.

### GWAS of 127 Peach Accessions

As shown in Figure 4B,C, we identified a total 19 SNPs above the threshold signal (1.2E-7). The most significant 7 SNPs were found in scaffold Pp06, and the most significant of all SNPs (3.4E-20) was at 26,924,482 bp of scaffold Pp06. This site is in the sixth intron of the gene *Prupe.6G292200*. Although 19 SNPs were above the threshold, only the single SNP at 26,924,482 bp of scaffold Pp06 was found in an interval (26.7 M∼27.2 M) reported by a previous linkage analysis study to be within an S-locus. In 11 out of 12 (91.7%) flat accessions in this GWAS population, this SNP is A/T (10/11) or T/T (1/11), while in all round accessions (93 accessions), this SNP was A/A.

### Local Manhattan Plot of the Most Associated SNPs

As shown in Figure 4D, we only found SNPs above the threshold signal within 200k intervals surrounding a SNP at 26,924,482 bp of scaffold Pp06. To our surprise, the LD pattern of SNPs within the interval surrounding the SNP at 26,924,482 bp of scaffold Pp06 showed relatively weak LD values; this was especially true for SNPs very near to SNP at 26,924,482 bp of scaffold Pp06.

### Validation of the Most Associated SNP in Additional Peach Accessions and Prunus Species

We further genotyped 258 additional peach accessions at this SNP and found that all 236 round-typed peach accessions carried an A/A genotype, while 22 flat-typed peach accessions carried an A/T genotype (Supplementary Table S6, Supplementary Data Sheet S3). Because the flat fruit shape has been observed only in peach, if the SNP at 26,924,482 bp of scaffold Pp06 is in fact a marker of the fruit shape S-locus, then a T allele at this SNP may not be found in other Prunus species. To test this prediction, we genotyped this SNP in 141 Prunus species available in the NCBI SRA dataset. We found that all Prunus species we genotyped at this SNP showed the A/A genotype. These results further supported the idea that SNP at 26,924,482 bp of scaffold Pp06 is closely related to peach fruit shape variance.

## Discussion

Sport mutations are common in many plant species including fruit trees, and are important for plant breeders because they provide novel variants for selection. Although previous works proposed that bud sports might occur at both the chromosome and gene levels (Foster and Aranzana, 2018), the precise molecular mechanisms responsible for bud sports – including those responsible for fruit shape bud sports – remain unknown. In the present study, a complex and strict SNP filter was used to produce a high-quality SNP set with a low error rate. This rate was two orders of magnitude lower than that observed in another peach sequencing study (Verde et al., 2013), and three orders of magnitude lower than rates observed in analogous studies of maize (Gore et al., 2009), chicken (Rubin et al., 2010), and rice (Huang et al., 2012; Xu et al., 2012). The total number of SNPs was smaller than in a previous study (Verde et al., 2013), suggesting that many previous identified SNPs may be artifacts of low-quality data processing. Using this high-quality SNP set, we identified a single, long LOH event in the bud sport genome that may be responsible for the fruit shape transition of flat peach from flat to round. The haplotype carrying the gene or genes determining the flat fruit trait was lost, leading the transition to the round shape. This conclusion was supported by four lines of evidence. First, the identified LOH event was responsible for the most significant differences between the wild-type and bud sport genomes (Figure 2A–C and Table 3). Second, this LOH event resulted in the loss of one of two haplotypes of the wild-type at the distal end of scaffold Pp06 (Figure 3). Third, our GWAS results located the gene or genes determining the flat fruit phenotype in the middle of this LOH interval. In addition, the GWAS also resolved which haplotype is responsible for the flat fruit character, by identifying the SNP site with the strongest association signal (Figure 4C). Fourth, the S-locus for peach fruit shape identified by a previous linkage analysis (Dirlewanger et al., 2006) was located within the middle of this LOH interval (Figure 2B). Taken together, we speculate that there are two main reasons why fruit shape alteration was likely derived from a single LOH event. First, bud sport occurrence was likely a low probability event because only one such specimen was found in the peach orchard examined in this study (Figure 1). Second, this bud sport occurred rather than at whole genome scale (Figure 2A, 3 and Table 2). Thus, the recombination of the two haplotypes requires a coincidental occurrence of at least two independent mutation events. A LOH event over a section of the genome is qualitatively different from others that occur at the whole genome level, as found in various types of cancer cells and model plants. This difference may be partly attributed to differences of in cellular regulation: cancer cells are deregulated at cell cycle, but flat peach cells are not.

Although, the SNP discovered by our GWAS was the same locus that was reported by a previous study (Cao et al., 2016), there were some differences between it and the current study. First, although our study included most of the peach accessions used in the previous study, 12 wild accessions, and 2 cultivars used in the previous work were not included here. Moreover, here we include 10 cultivars that were not included in the previous study. However, all the accessions used in the current study are cultivars, and therefore represent a less structured population. Second, the current study uses a different data processing and SNP calling pipeline than the previous study, and therefore resulted in a different SNP set. Third, a different statistical method was used to test the relationship between genotype and fruit shape. Fourth, we found that all round accessions showed an A/A genotype at 26,924,482 bp of scaffold Pp06, while all flat peach accessions but one showed A/T or T/T genotypes. This shows that all accessions with a T base at this locus are flat peach accessions, and thus the haplotype carrying T base is dominant and determines flat shape trait in peach. Fifth, despite the fact that the new peach accessions and *Prunus* species showed different results, all of them further validated our haplotype phasing result at 26,924,482 bp of scaffold Pp06. The haplotype difference at this SNP may be due to different uses of the reference genome sequence – i.e., the previous study (Cao et al., 2016) may use the negative strand as the reference sequence.

**FIGURE 5:**
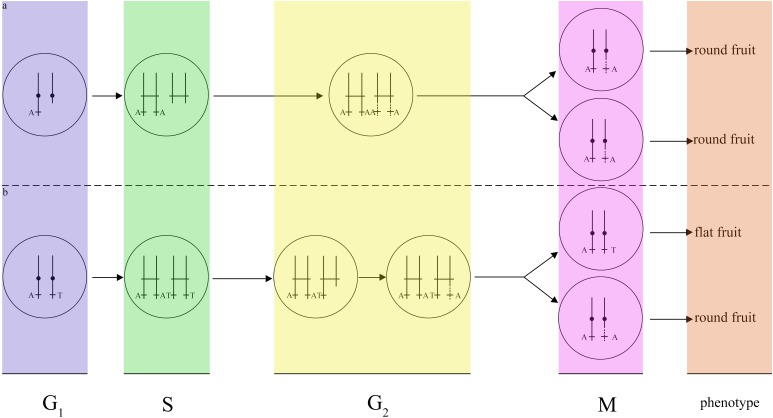
Models of two possible mechanisms for the fruit shape LOH event. **(a)** Break-induced replication in G1. If DNA damage occurs in G1, the cell will repair the DNA using a homologous chromosome in G2, resulting in only one type of daughter cell with the same round-type fruit phenotype. **(b)** Break-induced replication in G2. If DNA lesions occurred in G2, the cell will repair the DNA using a homologous chromosome in G2, and resulting in two types of daughter cells, one of which will have a round-type fruit phenotype and the other of which will have a flat-type fruit phenotype.

Although a variety of mechanisms putatively responsible for LOH events have been proposed (e.g., hemizygosity), LOH events in different cell types may result from distinct mechanisms. According to histogram cell theory (Satina et al., 1940), the peach mesocarps result from L-II cells. This LOH event is therefore explained by mitotic recombination, as suggested by previous works (Gisler et al., 2002). A DNA lesion may occur in G1. In this case, breakage would have occurred near ∼22 Mb of scaffold Pp06 in G1, and the lesion may not have been repaired by the cell. The broken DNA would then reduplicate as normal and enter G2, upon which both broken chromatids could have been repaired using the homologous scaffolds as templates. This model would result in a single type of cell that is homozygous for all markers from the breakage site to the end of scaffold Pp06 (Figure 5a). Another possibility is a break-induced replication in G2 near ∼22 Mb of scaffold Pp06; this model will cause two classes of cell. One would be heterozygous for all markers from the breakage site to the end of scaffold Pp06, while the other would be homozygous for these markers (Figure 5b). As the cell type of the bud sport in this study could not be definitively determined, we could not precisely identify the mechanism of this LOH event.

In conclusion, a long LOH event in a flat fruit haplotype in peach was identified in this study; this LOH event may therefore be responsible for the transition from the flat fruit wild-type shape to a round shape.

## Author Contributions

DG conceived and designed the experiments, revised the intellectual content of the manuscript, and supervised the project by correspondence. QT, XL, and HG performed the experiments and analyzed the data. WX, XC, XF, LL, DL, and DG provided technical and theoretical support to the manuscript. QT wrote the manuscript.

## Conflict of Interest Statement

The authors declare that the research was conducted in the absence of any commercial or financial relationships that could be construed as a potential conflict of interest.

## Footnotes

1 https://broadinstitute.github.io/picard/

2 http://www.ebi.ac.uk/

3 https://www.rosaceae.org/

4 http://www.novocraft.com/products/novoalign/

5 https://www.ncbi.nlm.nih.gov/bioproject/PRJNA487730

## Supplementary Material

METHODS Brief experiment methods were described for small Indel and large DEL variant calling pipeline, for the validation of origin of the homozygous haplotype in bud sport and for haplotype analysis at SNP 26,924,482 bp of scaffold Pp06 in additional peach accessions.

FIGURES S1–S5 Theses 5 figures represent the phenotype of bud sport and wild-type (Supplementary Figure S1), a LOH event supporting by small Indel (Supplementary Figure S2) and large DEL variants (Supplementary Figure S3), and a zoom IGV plot of small Indel (Supplementary Figure S4) and large DEL variant (Supplementary Figure S5), respectively.

TABLE S1 This result was obtained by vcftools with command -LROH under default parameter. This command was run for each scaffold of 3 samples vcf file (SAMN01000702, flattype, and roundtype), respectively. This result was the combination of 8 main scaffold.

TABLE S2 SRA run accessions of wild-type and bud sport, 23 low sequencing depth of SRA runs from GDR and 222 additional SRA runs from EBI were collected to conduct haplotype estimation.

TABLE S3 The above 247 SRA runs were integrated into 157 samples on the samples identity. The runs with the same sample identity were merged one sample.

TABLE S4 The above 157 samples were further filtered on PCA analysis and only the 127 cultivated peach accessions were kept for GWAS experiment.

TABLE S5 Additional 258 cultivated peach samples were phased at most association of SNP 26924482 bp of scaffold Pp06 from GWAS.

TABLE S6 In total 141 SRA runs from other *prunus* species excluding *prunus persica* were genotyped at most association of SNP 26924482 bp of scaffold Pp06 from GWAS.

TABLE S7 A first pair of primer was used to phase in additional 258 cultivated peach samples, and the rest primer pairs were to validate the origin of homozygous haplotype in bud sport.

DATA SHEET S2 The sanger sequencing of PCR product amplified from 7 pair of primers throughout distal end of scaffold Pp06.

DATA SHEET S3 The sanger sequencing of PCR product amplified from CAD_F/CAD_R primer pair in 258 peach accessions.
