# Corrigendum: Comparison Between Flat and Round Peaches, Genomic Evidences of Heterozygosity Events

**DOI:** 10.3389/fpls.2019.00929

**Published:** 2019-07-24

**Authors:** Qiuping Tan, Xiao Liu, Hongru Gao, Wei Xiao, Xiling Fu, Ling Li, Dongmei Li, Xiude Chen, Dongsheng Gao

**Affiliations:** ^1^College of Horticulture Science and Engineering, Shandong Agricultural University, Tai'an, China; ^2^State Key Laboratory of Crop Biology, Shandong Agricultural University, Tai'an, China; ^3^Shandong Collaborative Innovation Center for Fruit & Vegetable Production With High Quality and Efficiency, Shandong Agricultural University, Tai'an, China; ^4^College of Horticulture, Nanjing Agricultural University, Nanjing, China

**Keywords:** *Prunus persica*, fruit shape, bud sport, genome-wide association study, next generation sequencing

There is an error in the **Funding** statement. The correct number for the National Natural Science Foundation of China should only include 31872041.

We also neglected to include the funder the Natural Science Foundation of Shandong Province, ZR2018MC023 to Ling Li.

The correct **Funding** statement appears below:

This work was supported in part by a grant to DG from the National Natural Science Foundation of China (Grant No. 31872041), a grant to LL from the Natural Science Foundation of Shandong Province (Grant Nos. ZR2018MC023), the Ministry of Science and Technology of the People's Republic of China, and also, in part, supported by Fruit Innovation of Modern Agricultural Industry Technology System in Shandong Province (SDAIT-06-01).

Furthermore, in the published article there was an error in affiliation “3”. Instead of “Fruit Innovation of Modern Agricultural Industry Technology System in Shandong Province, Shandong Agricultural University, Tai'an, China”, it should be “Shandong Collaborative Innovation Center for Fruit & Vegetable Production with High Quality and Efficiency, Shandong Agricultural University, Tai'an, China”.

The original order of the authors was also incorrect. The original author was as follows: Quiping Tan, Xiao Liu, Hongru Gao, Wei Xiao, Xiude Chen, Xiling Fu, Ling Li, Dongmi Li, and Dongsheng Gao.

The correct author order is: Qiuping Tan, Xiao Liu, Hongru Gao, Wei Xiao, Xiling Fu, Ling Li, Dongmei Li, Xiude Chen, and Dongsheng Gao.

Additionally, in the original article only Dongsheng Gao was listed as a corresponding author. However, a correction has been made to include Xiude Chen as a corresponding author.

We also neglected to mention “Xiude Chen” in the author contribution statement of the published article. A correction has therefore been made to the **Author Contributions** statement:

DG and XC conceived and designed the experiments, revised the intellectual content of the manuscript, and supervised the project by correspondence. QT, XL, and HG performed the experiments and analyzed the data. WX, XF, LL, DL, XC, and DG provided technical and theoretical support to the manuscript. QT wrote the manuscript.”

Additionally, in the original article, there was an error. The novoalign and Varscan software parameters used were incorrectly formatted.

Corrections have been made to the **Materials and Methods**, subsection **Haplotype Construction Using the Whole-Genome SNP Set**, paragraph two:

Novoalign -d Prunus_persica_v2.0.a1_scaffolds -t 15, 3 -H 20 -softclip 20 -r Random -hlimit 8 -p 5, 20 -matchreward 3 -k -a GATCGGAAGAGCGGTTCAGCAGGAATGCCGAG; ACACTCTTTCCCTACACGACGCTCTTCCGATCT and the **Materials and Methods**, subsection **SNP Calling for GWAS**, paragraph two:

samtools mpileup -B -q 1 | java -jar varscan mpileup2snp -min-coverage 2 -min-reads2 1 -min-avg-qual 15 -min-var-freq 0.25 -min-freq-for-hom 0.75 -p-value 0.99 -output-vcf 1.

Lastly, there was a mistake in the supplementary table description for **Table S5** and **Table S6** as published. The correct table descriptions appear below.

“**Table S5** | Additional 258 cultivated peach samples were phased at most association of SNP 26,924,482 bp of scaffold Pp06 from GWAS.”

“**Table S6** | In total 141 SRA runs from other prunus species excluding prunus persica were genotyped at most association of SNP 26,924,482 bp of scaffold Pp06 from GWAS.”

The authors apologize for these errors and state that they do not change the scientific conclusions of the article in any way.

