# Supplementary material for: Comparison Between Flat and Round Peaches, Genomic Evidences of Heterozygosity Events
Source: Front Plant Sci. 2019 May 14;10:592. doi: 10.3389/fpls.2019.00592 (PMC6535965; doi:10.3389/fpls.2019.00592)
Supplement: METHODS — Brief experiment methods were described for small Indel and large DEL variant calling pipeline, for the validation of origin of the homozygous haplotype in bud sport and for haplotype analysis at SNP 26,924,482 bp of scaffold Pp06 in additional peach accessions. [file Data_Sheet_1.PDF]

Supplementary Methods

<1> Small indel calling

Small indel was called with GATK 3.8 pipeline, and filtered with hard filters “QD<2.0||FS>200.0|| ReadPosRankSum<-20.0”and bi-alleles were retained, the resulting filtered indel set was further filtered with vcftools (GQ<20, Max-missing=1, minDP=10, maxDP=45). The final indel set was used to extract genotype indivually for wild-type and bud sport and the genotype 0/0 was transferred to 0, 0/1 to 1, and 1/1 to 2 for R plot input.

<2> Large structural variant calling

Large structural variance was called both Pindel and Manta with default parameters. We chose the large DEL (>50bp) to analysis because of the relative easier discovering and genotyping. And variances supported with 5 reads were kept using Pindel tools. Meanwhile, average coverage depth of 3 samples was calculated with vcftools to determine the reasable depth of 20~70. All variance beyond this depth range were excluded. Variants called with manta diploid model were used for downstream analysis. Only common DEL variances called by two approaches were extracted for wild-type and bud sport mutant. The genotype recode was the same as that used in Indel variant.

<3>The origin of haplotye of bud sport

7 pair of primers were designed to validate that the homozygous distal end of scaffold Pp06 in bud sport mutant was derived from one of two haplotypes of wild-type. These primer pairs were evenly distributed in the distal end of scaffold Pp06. And each one of primer pair was in the DEL structure variant in wild-type, so the PCR was to amplify only one haplotype of wild-type. Theses PCRs were only conducted in wild-type, owing to only a single haplotype in bud sport at distal end of scaffold Pp06.

<4>Haplotype analysis in additional peach accessions

We design a pair of primer (CAD_F/CAD_R) to haplotype additional 258 peach cultivated accessions at SNP 26924482 bp of scaffold Pp06. The PCR products were validated with sanger sequencing.

Supplementary_Figures

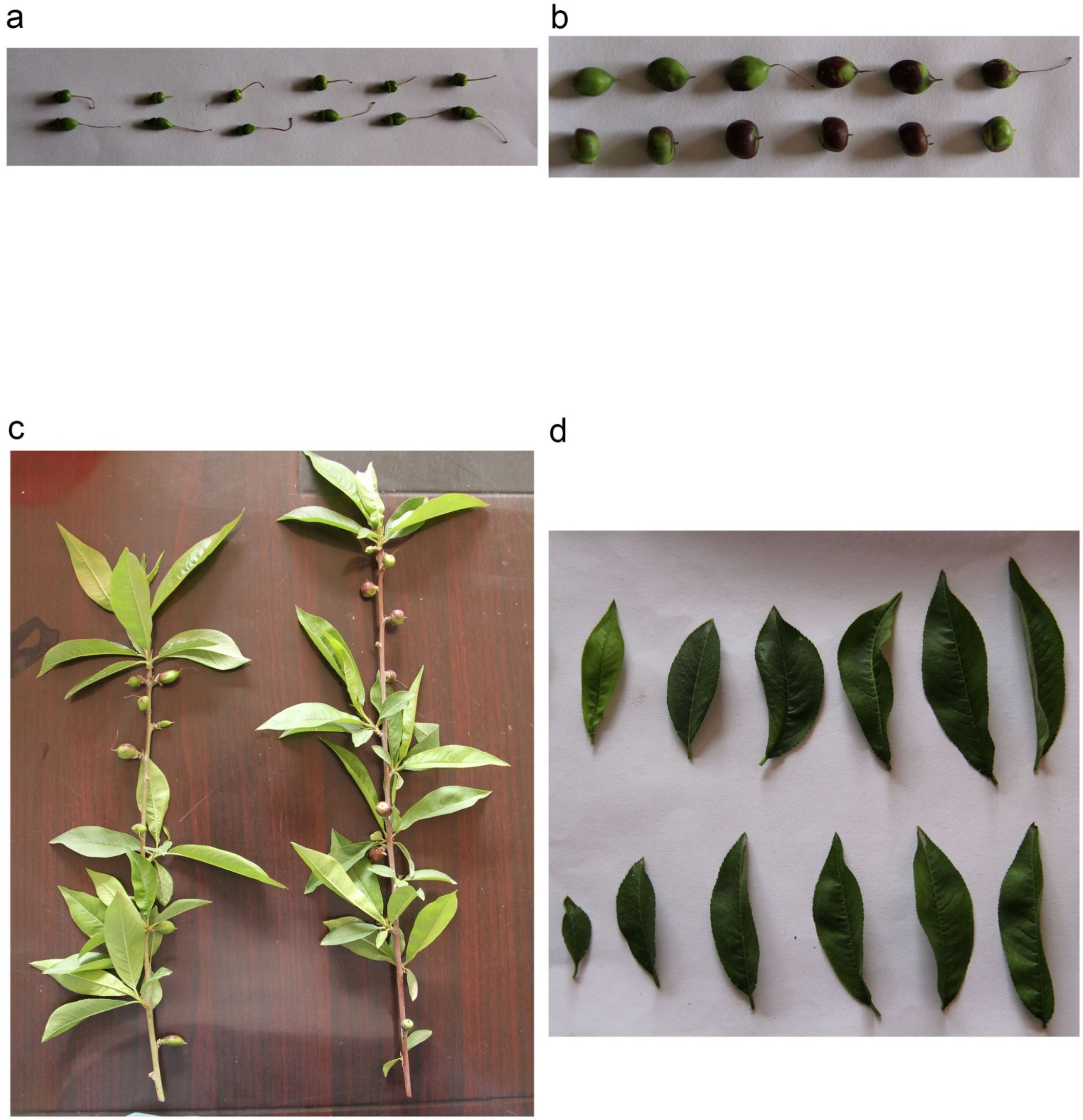

Fig S1 The phenotypic characterization of the flat peach and its bud sport. (a) The fruits from wild-type flat peach (top) and from bud sport (bottom) were photoed at about 10

days after flowering; (b) The fruits from wild-type flat peach (bottom) and from bud sport (top) were photoed at about 30 days after flowering; (c) The shoot from wild-type flat peach (right) and from bud sport (left) was photoed at 30 days after flowering; (d) The leave from wild-type flat peach (top) and from bud sport (bottom) were photoed at about 30 days after flowering.

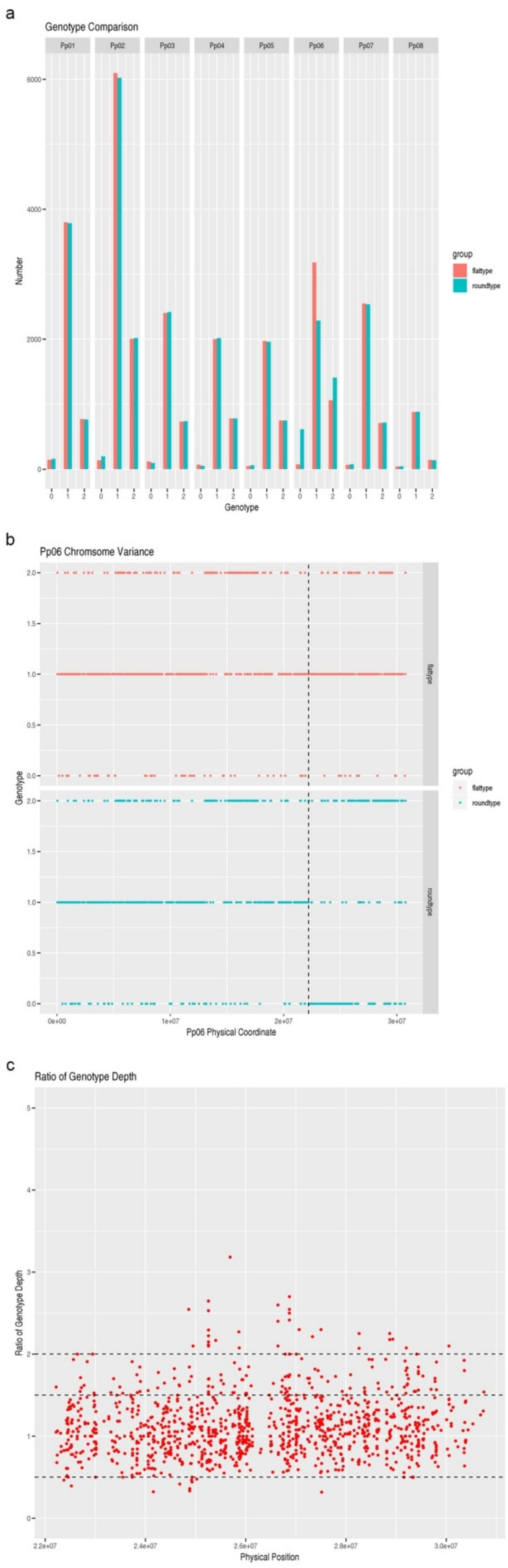

Fig S2 A LOH event occurring at the distal end of scaffold Pp06 of bud sport genome using Indel variant as marker

(a) The number of genotypes in each scaffold between wild-type and bud sport; (b) The distribution of genotype in scaffold Pp06 between wild-type and bud sport; (c)The distribution of depth ratio of genotypes at the distal end of scaffold Pp06 between wild-type and bud sport.

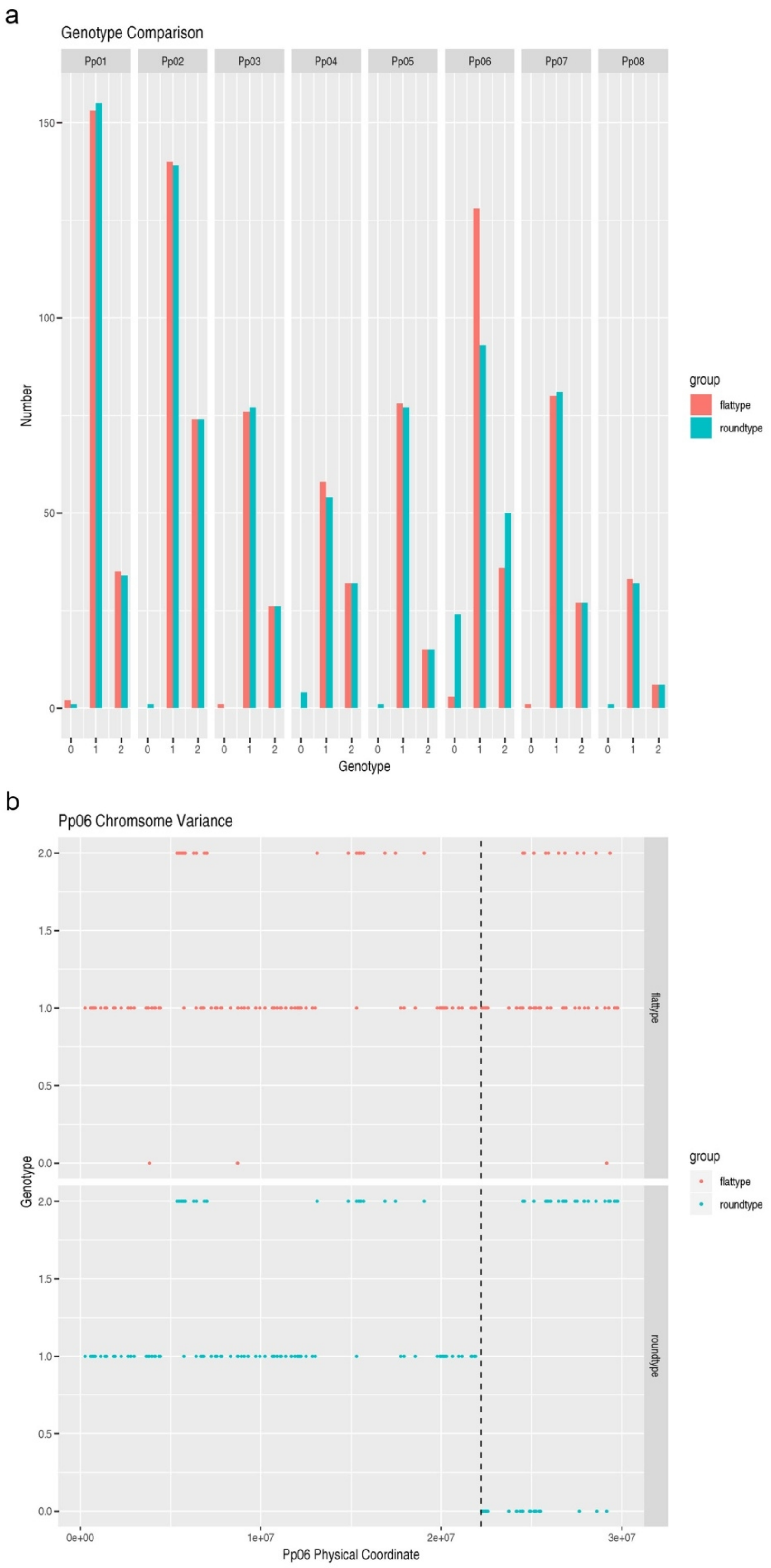

Fig S3 A LOH event occurring at the distal end of scaffold Pp06 of bud sport genome using large DEL variant as markers

(a) The number of genotypes in each scaffold between wild-type and bud sport; (b) The distribution of genotype in scaffold Pp06 between wild-type and bud sport;

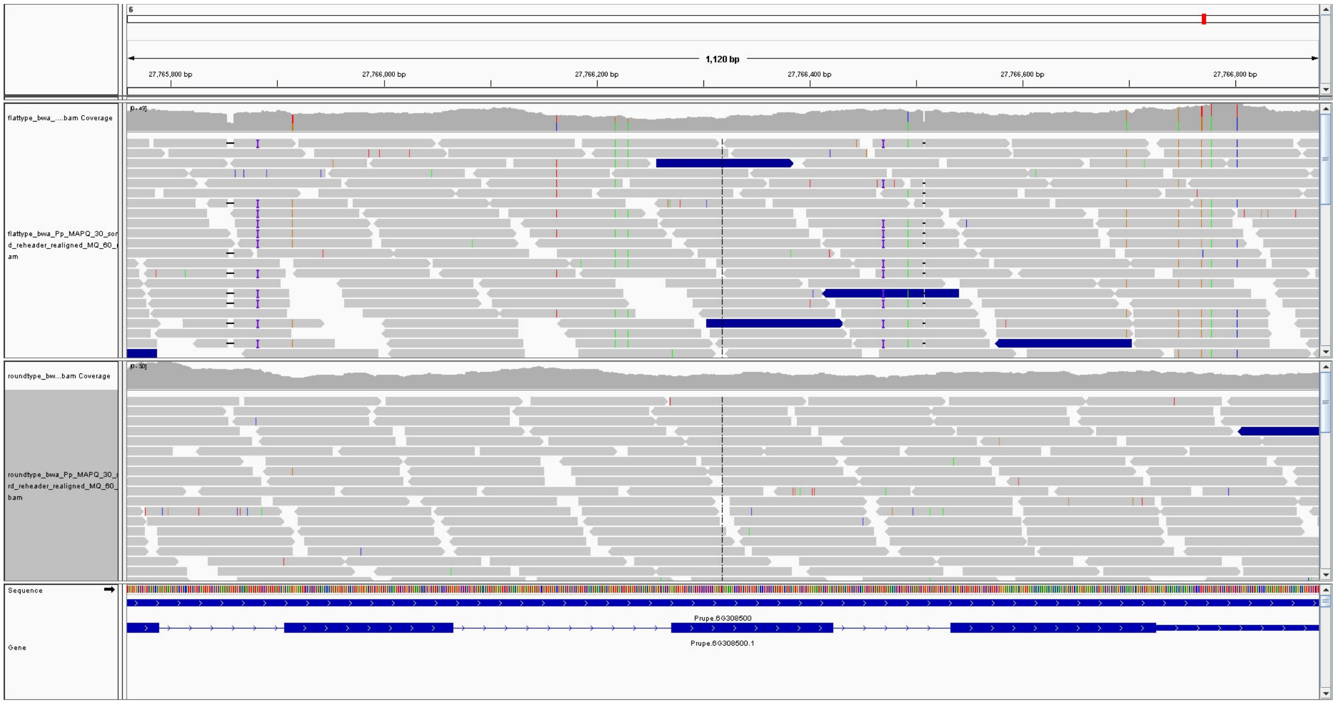

Fig S4 A LOH event occurring at the distal end of scaffold Pp06 of bud sport genome using SNP and small Indel as markers

Some SNP and small Indel variant were in wild-type (top panel) but was absent in bud sport (bottom panel). The bud sport had two copy of the same haplotype from reference genome.

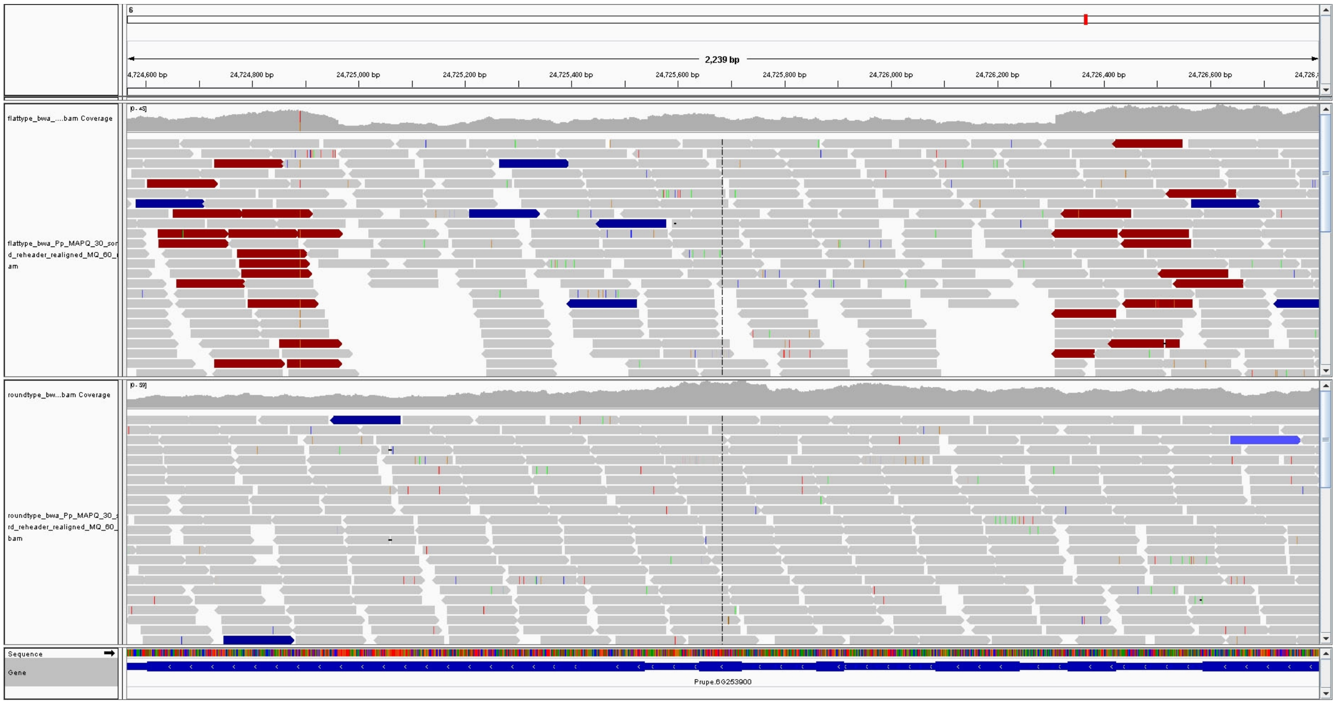

Fig S5 A LOH event occurring at the distal end of scaffold Pp06 of bud sport genome using a large DEL variant as marker

A large DEL variant was in wild-type (top panel) but was absent in bud sport (bottom panel). The bud sport had two copy of the same haplotype from reference genome.

Supplementary_Tables

Table S1 A LOH event detected by software vcftools at whole genome level

CHROM	AUTO_START	AUTO_END	MIN_START	MAX_END	N_VARIANTS_BETWEEN_MAX_BOUNDARIES	N_MISMATCHES	INDV
Pp01	2900	9677526	2901	14418733	11610	0	SAMN01000702
Pp01	9770005	9770005	2901	14418733	17274	0	SAMN01000702
Pp01	9995785	9995785	2901	14418733	22855	0	SAMN01000702
Pp01	10914172	14253115	2901	14418733	27833	0	SAMN01000702
Pp01	14487354	25911480	14418785	26056460	9975	0	SAMN01000702
Pp01	26454646	36230119	26056462	39149844	7953	2	SAMN01000702
Pp01	36532130	39084847	26056462	39149844	8691	0	SAMN01000702
Pp01	39175415	39175415	39149846	39403967	5	0	SAMN01000702
Pp01	39542606	41231690	39404026	41309592	900	0	SAMN01000702
Pp01	41312026	47850359	41309594	47850359	4840	0	SAMN01000702
Pp02	34586	1009234	34587	1696580	5835	0	SAMN01000702
Pp02	1990016	5111709	1696626	9403645	26172	0	SAMN01000702
Pp02	5787618	8409834	1696626	9403645	33828	0	SAMN01000702
Pp02	9533302	18734237	9404181	18745079	21076	1	SAMN01000702
Pp02	18914384	19331425	18886932	19584381	1262	0	SAMN01000702
Pp02	19717734	20929199	19584383	24133052	5874	0	SAMN01000702
Pp02	21348273	23704278	19584383	24133052	9044	0	SAMN01000702
Pp02	24139024	30096867	24135780	30344165	3063	0	SAMN01000702
Pp03	395	5547420	396	5597382	4152	1	SAMN01000702
Pp03	5826939	27031263	5597384	27326548	19128	0	SAMN01000702
Pp04	58274	4611064	58275	4625642	3350	0	SAMN01000702
Pp04	4628156	5686298	4625746	10809932	5965	0	SAMN01000702
Pp04	6272067	10252076	4625746	10809932	9453	0	SAMN01000702
Pp04	11575556	14177393	10809939	14181658	4646	0	SAMN01000702
Pp04	14188967	16868469	14183342	16881899	3918	1	SAMN01000702
Pp04	16883746	16883906	16881901	16883937	3	0	SAMN01000702
Pp04	16892769	16892832	16883939	16894374	3	0	SAMN01000702
Pp04	16896599	22345346	16895244	25511242	3930	3	SAMN01000702
Pp04	22594941	22598179	16895244	25511242	4773	0	SAMN01000702
Pp04	22697814	22697814	16895244	25511242	5613	0	SAMN01000702
Pp05	104490	4504101	104491	18364439	24476	0	SAMN01000702
Pp05	5357646	14409431	104491	18364439	35022	0	SAMN01000702
Pp05	16186530	16369290	104491	18364439	35313	0	SAMN01000702
Pp05	17623382	18232588	104491	18364439	35349	0	SAMN01000702
Pp06	6768	5742559	6769	5811433	5781	0	SAMN01000702
Pp06	5746500	5746500	6769	5811433	5862	0	SAMN01000702
Pp06	6030646	6628209	5811445	6628436	752	0	SAMN01000702
Pp06	6628602	6628602	6628438	6628780	1	0	SAMN01000702
Pp06	6628920	13017866	6628894	25364214	21658	0	SAMN01000702
Pp06	14827176	15571490	6628894	25364214	34204	0	SAMN01000702
Pp06	15761937	15761951	6628894	25364214	44400	0	SAMN01000702
Pp06	17398588	25363711	6628894	25364214	52694	0	SAMN01000702
Pp06	25365172	30691873	25364218	30691873	6866	0	SAMN01000702
Pp06	22215499	25363711	22195189	25364214	3632	1	roundtype
Pp06	25365172	25784958	25364218	25844683	460	0	roundtype
Pp06	25848223	26579670	25844686	26651259	696	2	roundtype
Pp06	26719756	28259534	26652362	28262267	2692	1	roundtype
Pp06	28265695	30363818	28262918	30384134	2917	3	roundtype
Pp07	11743	5233678	11744	15124182	22403	0	SAMN01000702
Pp07	5859771	21835883	11744	21835883	38858	1	SAMN01000702
Pp08	403290	22571751	209642	22571751	7100	3	SAMN01000702

Table S2 247 SRA run accessions for haplotype phasing

Rnu_ID
flattype
roundtype
Prunus_persica-Admiral_Dewey-DPRU1190_1
Prunus_persica-Admiral_Dewey-DPRU1190_2
Prunus_persica-Babcock_1
Prunus_persica-Babcock_2
Prunus_persica-Bolinha_1
Prunus_persica-Bolinha_2
Prunus_persica-Carmen-DPRU2142_1
Prunus_persica-Carmen-DPRU2142_2
Prunus_persica-Chinese_cling_1
Prunus_persica-Chinese_cling_2
Prunus_persica-Diamante_1
Prunus_persica-Diamante_2
Prunus_persica-Dixon_1
Prunus_persica-Dixon_2
Prunus_persica-Dr_Davis_1
Prunus_persica-Dr_Davis_2
Prunus_persica-Early_Crawford-DPRU0589_1
Prunus_persica-Early_Crawford-DPRU0589_2
Prunus_persica-Elberta_1
Prunus_persica-Elberta_2
Prunus_persica-Florida_Prince-P138_1
Prunus_persica-Florida_Prince-P138_2
Prunus_persica-Georgia_Bell_1
Prunus_persica-Georgia_Bell_2
Prunus_persica-JH_Hale_1
Prunus_persica-JH_Hale_2
Prunus_persica-Lovell_1
Prunus_persica-Lovell_2
Prunus_persica-Mayflower_1

Prunus_persica-Mayflower_2
Prunus_persica-Nemaguard_1
Prunus_persica-Nemaguard_2
Prunus_persica-Nonpareil_1
Prunus_persica-Nonpareil_2
Prunus_persica-OHenry_1
Prunus_persica-OHenry_2
Prunus_persica-Okinawa_1
Prunus_persica-Okinawa_2
Prunus_persica-Oldmixon_Free_1
Prunus_persica-Oldmixon_Free_2
Prunus_persica-Rio_Oso_Gem_1
Prunus_persica-Rio_Oso_Gem_2
Prunus_persica-Slappey-DPRU2179_1
Prunus_persica-Slappey-DPRU2179_2
Prunus_persica-St_John_Yellow-DPRU0941_1
Prunus_persica-St_John_Yellow-DPRU0941_2
SRR830496
SRR830497
SRR830498
SRR830499
SRR830500
SRR830501
SRR830502
SRR830503
SRR830504
SRR830505
SRR830506
SRR830507
SRR830508
SRR830509
SRR830510
SRR830511
SRR830512

SRR830513
SRR830514
SRR830515
SRR830516
SRR830517
SRR830518
SRR830519
SRR830520
SRR830521
SRR830522
SRR830523
SRR830524
SRR830525
SRR830526
SRR830527
SRR830528
SRR830529
SRR830530
SRR830531
SRR830532
SRR830533
SRR830534
SRR830535
SRR830536
SRR830537
SRR830538
SRR830539
SRR830540
SRR830541
SRR830542
SRR830543
SRR830544
SRR830545
SRR830546

SRR830547
SRR830548
SRR830549
SRR830550
SRR830551
SRR830552
SRR830553
SRR830554
SRR830555
SRR830556
SRR830557
SRR830558
SRR830559
SRR830560
SRR830561
SRR830562
SRR830563
SRR830564
SRR830565
SRR830566
SRR830567
SRR830568
SRR830569
SRR830570
SRR830571
SRR830572
SRR830573
SRR830574
SRR830575
SRR830576
SRR830577
SRR830578
SRR830579
SRR1994351

SRR1994352
SRR1994353
SRR1994354
SRR1994355
SRR1994356
SRR1994357
SRR1994358
SRR1994359
SRR1994360
SRR1994361
SRR1994362
SRR1994363
SRR1994364
SRR1994365
SRR1994366
SRR1994367
SRR1994368
SRR1994369
SRR1994370
SRR1994371
SRR1994372
SRR1994373
SRR1994374
SRR1994375
SRR1994376
SRR1994377
SRR1994378
SRR1994379
SRR1994380
SRR1994381
SRR1994382
SRR1994383
SRR1994384
SRR1994385

SRR1994386
SRR1994387
SRR1994388
SRR1994389
SRR1994390
SRR2056073
SRR2056074
SRR2056075
SRR2056076
SRR2056077
SRR2056078
SRR2056079
SRR2056080
SRR2056081
SRR2056082
SRR2056083
SRR2056084
SRR2056085
SRR2056086
SRR2056087
SRR2056088
SRR2056089
SRR2056090
SRR2056091
SRR2056092
SRR2056093
SRR2056094
SRR2056095
SRR2056096
SRR2056097
SRR2056098
SRR2056099
SRR2056100
SRR2056101

SRR2056102
SRR2056103
SRR2056104
SRR2056105
SRR2056106
SRR2056107
SRR2056108
SRR2056109
SRR2056110
SRR2056111
SRR2056112
SRR1867740
SRR1867794
SRR1867795
SRR1867806
SRR502985
SRR502994
SRR502993
SRR502992
SRR502991
SRR502990
SRR502989
SRR502987
SRR502986
SRR503001
SRR503000
SRR502983
SRR502997
SRR502996
SRR502995
SRR501836
SRR074976
SRR074977
SRR074951

SRR091233
SRR068366
SRR068367
SRR068368
SRR068369
SRR068361
SRR068359
SRR068362
SRR068363
SRR068364
SRR068365
SRR068360

Table S3 157 Samples ID integrated by 247 runs on sample identity

Samples_ID
flattype
roundtype
Prunus_persica-Admiral_Dewey-DPRU1190
Prunus_persica-Babcock
Prunus_persica-Bolinha
Prunus_persica-Carmen-DPRU2142
Prunus_persica-Diamante
Prunus_persica-Dixon
Prunus_persica-Early_Crawford-DPRU0589
Prunus_persica-Florida_Prince-P138
Prunus_persica-JH_Hale
Prunus_persica-Lovell
Prunus_persica-Mayflower
Prunus_persica-Nemaguard
Prunus_persica-OHenry
Prunus_persica-Okinawa
Prunus_persica-Oldmixon_Free
Prunus_persica-Rio_Oso_Gem

Prunus_persica-Slappey-DPRU2179
Prunus_persica-St_John_Yellow-DPRU0941
SAMN00115266
SAMN00115267
SAMN00115268
SAMN00139512
SAMN01000698
SAMN01000701
SAMN01000704
SAMN01000706
SAMN02048712
SAMN02048738
SAMN02048758
SAMN02048789
SAMN03393052
SAMN03565481
SAMN03565482
SAMN03565483
SAMN03565484
SAMN03565485
SAMN03565486
SAMN03565487
SAMN03565488
SAMN03565489
SAMN03565490
SAMN03565491
SAMN03565492
SAMN03565493
SAMN03565494
SAMN03565495
SAMN03565496
SAMN03565497
SAMN03565498
SAMN03565499

SAMN03565500
SAMN03565501
SAMN03565502
SAMN03565503
SAMN03565504
SAMN03565505
SAMN03565506
SAMN03565507
SAMN03565508
SAMN03565509
SAMN03565510
SAMN03565511
SAMN03565512
SAMN03565513
SAMN03565514
SAMN03565515
SAMN03565516
SAMN03565517
SAMN03565518
SRR501836
SRR502983
SRR502985
SRR502986
SRR502994
SRR502997
SRR830496
SRR830498
SRR830499
SRR830500
SRR830501
SRR830502
SRR830503
SRR830504
SRR830505

SRR830506
SRR830507
SRR830508
SRR830509
SRR830510
SRR830511
SRR830512
SRR830513
SRR830514
SRR830515
SRR830516
SRR830517
SRR830518
SRR830519
SRR830520
SRR830521
SRR830522
SRR830524
SRR830525
SRR830526
SRR830527
SRR830528
SRR830529
SRR830530
SRR830531
SRR830532
SRR830533
SRR830534
SRR830535
SRR830536
SRR830537
SRR830538
SRR830539
SRR830540

SRR830541
SRR830542
SRR830544
SRR830545
SRR830546
SRR830547
SRR830548
SRR830549
SRR830550
SRR830551
SRR830552
SRR830553
SRR830554
SRR830555
SRR830556
SRR830557
SRR830558
SRR830559
SRR830560
SRR830561
SRR830562
SRR830563
SRR830564
SRR830565
SRR830566
SRR830567
SRR830568
SRR830569
SRR830570
SRR830571
SRR830572
SRR830573
SRR830575
SRR830576

SRR830577
SRR830578
SRR830579

Table S4 127 cultivated peach samples used for GWAS on PCA analysis

Samples_Accession
flattype
roundtype
SAMN00115266
SAMN00115267
SAMN00115268
SAMN00139512
SAMN01000698
SAMN01000701
SAMN01000704
SAMN01000706
SAMN02048712
SAMN02048738
SAMN02048758
SAMN02048789
SAMN03393052
SAMN03565481
SAMN03565482
SAMN03565483
SAMN03565484
SAMN03565485
SAMN03565486
SAMN03565487
SAMN03565488
SAMN03565489
SAMN03565490
SAMN03565491
SAMN03565492

SAMN03565494
SAMN03565495
SAMN03565496
SAMN03565498
SAMN03565499
SAMN03565500
SAMN03565501
SAMN03565502
SAMN03565503
SAMN03565504
SAMN03565505
SAMN03565506
SAMN03565507
SAMN03565508
SAMN03565509
SAMN03565510
SAMN03565511
SAMN03565512
SAMN03565513
SAMN03565514
SAMN03565515
SAMN03565516
SAMN03565517
SAMN03565518
SRR501836
SRR502983
SRR502985
SRR502986
SRR502994
SRR502997
SRR830496
SRR830498
SRR830502
SRR830504

SRR830505
SRR830506
SRR830507
SRR830508
SRR830509
SRR830510
SRR830514
SRR830518
SRR830519
SRR830520
SRR830521
SRR830522
SRR830524
SRR830525
SRR830526
SRR830527
SRR830528
SRR830529
SRR830530
SRR830531
SRR830532
SRR830533
SRR830534
SRR830535
SRR830536
SRR830537
SRR830538
SRR830539
SRR830540
SRR830541
SRR830542
SRR830544
SRR830545
SRR830546

SRR830547
SRR830548
SRR830549
SRR830550
SRR830551
SRR830552
SRR830553
SRR830554
SRR830555
SRR830556
SRR830557
SRR830558
SRR830559
SRR830560
SRR830561
SRR830562
SRR830563
SRR830564
SRR830565
SRR830566
SRR830567
SRR830568
SRR830569
SRR830570
SRR830571
SRR830572
SRR830573
SRR830575
SRR830576
SRR830577
SRR830578
SRR830579

Table S5 Haplotype phasing SNP at 26924482 bp of scaffold Pp06 in additional 258 cultivated peach accessions

Sample_ID	Genotype	Fruit_shape
No_34_1	A/A	Round
No_34_2	A/A	Round
You_Xi_F	A/A	Round
No_8	A/A	Round
12_33	A/A	Round
Li_He_You_Ming	A/A	Round
Zao_Shu_You_Ming	AA	Round
No_12	A/A	Round
No_47	A/A	Round
Zhao_Xia	A/A	Round
Chun_Jie	A/A	Round
Qiu_Tong	A/A	Round
No_13_1	A/A	Round
Zao_No_4	A/A	Round
No_1	A/A	Round
Zhong_You_No_20	A/A	Round
Bei_Jing_No_8	NA	NA
Jin_Mi_Xia_Ye	A/A	Round
Qiu_Xue	A/A	Round
Hong_Se_Ma_Li_Ya	A/A	Round
No_39_13_57	A/A	Round
Gui_Yu_5_48	A/A	NA
Xia_Tian_No_1	A/A	Round
Ju_Xuan_Te_Zao_Mi	A/A	Round
Xia_Li	A/A	NA
Zao_Pan_Tao	A/T	Flat
No_25	A/A	Round
Hong_Xing_No_4	A/A	Round
99_Nan_5_5	A/A	Round
No_23_24	A/A	Round
No_13_12	A/A	Round
Ji_Zao_Shu_Tao	A/A	Round

No_13_21	A/A	Round
Mei_Jia_No_1	A/A	Round
Zi_Pan_Tao	A/T	Flat
Tian_Huang_Tao_8_Yue_Shang	A/A	Round
Huang_Rou_You_Tao_No_1	A/A	Round
No_13_9_5	A/A	Round
No_13_29	A/A	Round
Xin_7_13_30	A/A	Round
Xia_Xue	A/A	Round
No_13_6	A/A	Round
Xin_19_13_44_No_1	A/A	Round
Song_Tao	A/A	Round
Zhong_Qiu_Dong_Tao	A/A	Round
Pan_Tao_8_Yue	A/T	Flat
No_20	A/A	Round
Hong_Jiu_Xian	A/A	Round
No_13_65	A/A	Round
No_18_13_47	A/A	Round
No_13_50	A/A	Round
Zhong_Zhou_Dao_No_2	A/A	Round
Huang_Tao	A/A	Round
Qi_Yue_You_Pan_No_1	A/A	Round
No_19	A/A	Round
Unkown	A/A	Round
Tian_Huang_Tao_8_Yue_Zhong_No_1	A/A	Round
Qi_Yue_You_Pan_No_2	A/A	Round
Shi_Wu_Hong_Tao	A/A	Round
No_13_5	A/A	Round
No_13_62	A/A	Round
No_13_11_9	A/A	Round
No_13_66	A/A	Round
No_13_20_110	A/A	Round
No_9_Ya_Bian	A/A	Round
Zhong_Hua_Wan_Cui_Tao	A/A	Round

Huang_Rou_You_Tao_No_2	A/A	Round
Zhong_You_No_19	A/A	Round
Hong_You_Pan_7_Yue_Zhong	A/T	Flat
Ji_Zao_Hong_You	A/A	Round
Wan_You_Tao	A/A	Round
Xin_Zhou_Zhong_Dao	A/A	Round
No_13_58	A/A	Round
Zao_You_Tao_Shi_Sheng	A/A	Round
No_13_49	A/A	Round
Wan_You_Tao_No_48	A/A	Round
Peng_Xian_No_14	A/A	Round
Peng_Xian_No_8	A/A	Round
Wan_Cui_Hong	A/A	Round
Gan_Hong_No_1	A/A	Round
Li_De_Hua_No_502	A/A	Round
Hua_Guo_Jian_Yong_You_Tao	A/A	Round
Shen_Yang_Kang_Han_Mao_Tao	A/A	Round
No_98_6_48	A/A	Round
Hong_Qing_Shui_No_2	A/A	Round
Zhong_Hua_Fu_Tao	A/A	Round
Tian_Huang_Tao_8_Yue_Zhong_No_2	A/A	Round
Nai_Hu_nei	A/A	Round
Bai_Rou_You_Tao	A/A	Round
Da_Guo_You_Gao_Tang_8_Yue	A/A	Round
No_13_37	A/A	Round
Fang_Cheng_Wan_Mi	A/A	Round
You_Pan_Tao	A/T	Flat
No_98_5_59	A/A	Round
Bian_You_Tao	A/T	Flat
Qiu_Wang_You_Tao	A/A	Round
No_27	A/A	Round
Sha_Piao_Hong	A/A	Round
No_6	A/A	Round

Xin_19_13_44_No_2	A/A	Round
Jin_Yu_You_Tao	A/A	Round
Hua_Guo_Jian_Yong_You_Tao_Zhu	A/A	Round
Suan_You_Tao	A/A	Round
Wan_Cang_Fang_Zao_Sheng	A/A	Round
Zi_Xue_Hong_Tao	A/A	Round
Han_Guo_Da_Bai_Tao	A/A	Round
Qiu_Tao	A/A	Round
Mao_Tao_9_Yue	A/A	Round
Hong_Bai_Hua_Tao	A/A	Round
Huang_Pan_Tao_No_19	A/A	Round
Bing_Tang_Hong	A/A	Round
Huang_Jin_Mi_No_3	A/A	Round
Jin_Shuo	A/A	Round
Ju_Pan_Tao	A/T	Flat
Hong_Zhi_Guo	A/A	Round
No_13_32	A/A	Round
Xin_No_4	A/A	Round
Tian_Huang_Tao_7_Yue_Shang	A/A	Round
Bai_Tao_6_Yue	A/A	Round
No_13_43	A/A	Round
Xin_6_13_22	A/A	Round
Mao_Pan_7_Yue_Zhong_Xia	A/T	Flat
No_9_Ya_Bian_Ying	A/A	Round
Ya_Yong_Wan_Tao_9_Yue	A/A	Round
No_13_39	A/A	Round
Unkown_2	A/A	Round
No_13_24	A/A	Round
No_13_27	A/A	Round
Xin_13_44	A/A	Round
Long_Zhu_Bi_Tao	A/A	Round
No_14_1_40	A/A	Round
Xin_14_13_38	A/A	Round
Chong_Yang_Hong	A/A	Round

Shi_Yue_Hong_Tao	A/A	Round
No_13_65_18	A/A	Round
Da_Pan_Tao_8_Yue_Zhong	A/T	Flat
Chao_Hong_Mao	A/A	Round
No_43	A/A	Round
He_Bei_Tie_Tao	A/A	Round
No_13_2	A/A	Round
Hua_Guo_Jian_Yong_You_Tao_Fen	A/A	Round
No_13_16	A/A	Round
No_13_10_41	A/A	Round
No_13_2_3	A/A	Round
Huang_Tao_No_83	A/A	Round
You_Pan_Tao_7_Yue_Shang_No_1	A/T	Flat
Hong_Hua_Mao_Tao	A/A	Round
Ping_Lu_Hong_Bu_Ruan	A/A	Round
Mao_Pan_Tao	A/T	Flat
No_13_46	A/A	Round
Gan_Lu	A/A	Round
Mao_Pan	A/A	Round
Zhong_You_No_8	A/A	Round
Mao_Pan_Mao	A/A	Round
Xin_15_13_45	A/A	Round
No_13_13	A/A	Round
Fen_Hua_Hong_Tao	A/A	Round
Rui_Xiang_Huang_Tao	A/A	Round
No_9_Ya_Bian_Zao	A/A	Round
Zao_Shu_Tao_He_Nan	A/A	Round
You_Pan_6_Yue_Di	A/T	Flat
8_Yue_Pan_Tao	A/T	Flat
Ao_Hong_Cui_Zao	A/A	Round
Mao_Pan_7_Yue_Zhong	A/T	Flat
Jin_Qiu_Huang_Pan	A/T	Flat
No_9_Ya_Bian_Suan	A/A	Round

Ying_Shuang_Hong	A/A	Round
Zhao_Yang_You_Tao	A/A	Round
Zao_Shu_Huang_You-Tao	A/A	Round
Jia_Zhou_Zao_Tian	A/A	Round
Jin_Mo	A/A	Round
Chun_Guang_You_Tao	A/A	Round
Tian_Huang_Tao_7_Yue_Xia	A/A	Round
Zao_Sha_Piao_Hong	A/A	Round
You_Pan_Tao_7_Yue_Shang_No_2	A/T	Flat
Hong_Gan_Lu	A/A	Round
Da_Guo_Gao_Tang_You_9_20	A/A	Round
Gan_Su_You_Tao_No_5	A/A	Round
Gan_Su_You_Tao_No_1	A/A	Round
Gan_Su_You_Tao_No_17	A/A	Round
Gan_Su_You_Tao_No_8	A/A	Round
Gan_Su_You_Tao_No_4	A/A	Round
Gan_Su_You_Tao_No_15	A/A	Round
Gan_Su_You_Tao_No_16	A/A	Round
Gan_Su_You_Tao_No_2	A/A	Round
Gan_Su_You_Tao_No_3	A/T	Flat
Gan_Su_You_Tao_No_9	A/A	Round
Gan_Su_You_Tao_No_14	A/A	Round
Gan_Su_You_Tao_No_11	A/A	Round
Gan_Su_You_Tao_No_6	A/A	Round
Gan_Su_You_Tao_No_19	A/A	Round
Gan_Su_You_Tao_No_12	A/A	Round
Gan_Su_You_Tao_No_18	A/A	Round
Gan_Su_You_Tao_No_13	A/A	Round
Gan_Su_You_Tao_No_7	A/A	Round
Gan_Su_You_Tao_No_10	A/A	Round
Zi_Yan_Hong	A/A	Round
Qing_Wu_Pi	A/A	Round
Ao_Hong_Cui	A/A	Round
Tu_Wei	A/A	Round

Rui_Guang_No_28	A/A	Round
Chun_Xue	A/A	Round
Jin_Mei_Xia	A/A	Round
Jing_He_You_No_2	A/A	Round
Zhong_You_No_16	A/A	Round
Nan_Fang_Jin_Mi	A/A	Round
Rui_Hong	A/A	Round
Xia_Tian_No_2	A/A	Round
Hong_Qing_Shui	A/A	Round
Zao_Huang_Mi	A/A	Round
Pan_Tao_Wang	A/T	Flat
Xia_Xiang_Ji	A/A	Round
Bao_Ling_Mi	A/A	Round
Zhong_Hua_Hong_Mi_Tao	A/A	Round
Mei_Jia_No_2	A/A	Round
Mei_Jia_No_3	A/A	NA
7_Yue_You_Pan	A/A	Round
Mei_Qing_Bai_Tao	A/A	Round
Jing_He_You_No_1	A/A	Round
Li_Xiang	A/A	Round
Rui_Guang_No_11	A/A	Round
Yong_Lian_Mi_Tao	A/A	Round
Cang_Fang_Zao_Sheng	A/A	Round
Mei_Jia_No_4	A/A	Round
Jing_Xiu_Huang_Tao	A/A	Round
Fu_Dao_Tao_Wang	A/A	Round
Zao_Yu_Tao	A/A	Round
A_Bu_Bai_Tao	A/A	Round
Peng_Xian_No_7	A/A	Round
Zao_Chun_Mi	A/A	Round
Hong_Gan_Lu_Mao	A/A	Round
Rui_Pan_No_14	A/T	Flat
Rui_Guang_No_19	A/A	Round
Wang_Shou_Hong	A/A	Round

Xu_Ri	A/A	Round
Chao_Hong_Zhu	A/A	Round
Shi_Tou_Tao	A/A	Round
Du_Bin	A/A	Round
Zhong_You_No_12	A/A	Round
Huang_Jin_Mi_No_4	A/A	Round
Da_Tuan_Mi_Lu	A/A	Round
Hong_Gang_Shan	A/A	Round
Zao_Mei	A/A	Round
Wu_Yue_Yang_Guang	A/A	Round
Rui_Pan_No_4	A/T	Flat
Chun_Mi	A/A	Round
Xiu_Yu_You_Tao	A/A	Round
Jin_Tong_No_5	A/A	Round
Rui_Guang_No_29	A/A	Round
Xian_Dao_Ming_Zhu	A/A	Round
Mei_Jia_No_5	A/A	Round
Huang_Jin_Mi_No_2	A/A	Round
Ji_Zao_Pan	A/T	Flat
Chao_Zao_Hong	A/A	Round
Yi_Meng_Shuang_Hong	A/A	Round
Zao_Lu_Pan	A/T	Flat
Shu_Guang_You_Tao	A/A	Round
Wu_Yue_Xian_Huang_Tao	A/A	Round

Table S6 Genotyping SNP at 26924482 bp of scaffold Pp06 in 141 Prunus species

Species/Run_accession	Number/Genotype	Fruit_Shape
Prunus mume	13	NA
SRR5241555	A/A	Round
SRR5241554	A/A	Round
SRR5241553	A/A	Round
SRR5241552	A/A	Round
SRR5241550	A/A	Round

SRR5241549	A/A	Round
SRR540231	A/A	Round
SRR540230	A/A	Round
SRR531301	A/A	Round
SRR531299	A/A	Round
SRR531298	A/A	Round
SRR531296	A/A	Round
SRR654705	A/A	Round
Prunus dulcis	30	NA
SRR4045229	A/A	Round
SRR4045228	A/A	Round
SRR4045227	A/A	Round
SRR4045226	A/A	Round
SRR4045225	A/A	Round
SRR4045224	A/A	Round
SRR4045223	A/A	Round
SRR4036108	A/A	Round
SRR4036105	A/A	Round
SRR3141248	A/A	Round
SRR3141238	A/A	Round
SRR3141229	A/A	Round
SRR3141204	A/A	Round
SRR3141192	A/A	Round
SRR3141181	A/A	Round
SRR3141113	A/A	Round
SRR3141098	A/A	Round
SRR3141083	A/A	Round
SRR3141073	A/A	Round
SRR3141065	A/A	Round
SRR3141057	A/A	Round
SRR3141049	A/A	Round
SRR3141040	A/A	Round
SRR3141032	A/A	Round
SRR765861	A/A	Round

SRR765850	A/A	Round
SRR765838	A/A	Round
SRR765679	A/A	Round
Prunus armeniaca	66	NA
SRR2153164	A/A	Round
SRR2153135	A/A	Round
SRR2165084	A/A	Round
SRR2165083	A/A	Round
SRR2153139	A/A	Round
SRR2153192	A/A	Round
SRR2153191	A/A	Round
SRR2153190	A/A	Round
SRR2153189	A/A	Round
SRR2153188	A/A	Round
SRR2153187	A/A	Round
SRR2153186	A/A	Round
SRR2153185	A/A	Round
SRR2153184	A/A	Round
SRR2153183	A/A	Round
SRR2153182	A/A	Round
SRR2153181	A/A	Round
SRR2153180	A/A	Round
SRR2153179	A/A	Round
SRR2153178	A/A	Round
SRR2153177	A/A	Round
SRR2153176	A/A	Round
SRR2153175	A/A	Round
SRR2153174	A/A	Round
SRR2153173	A/A	Round
SRR2153172	A/A	Round
SRR2153171	A/A	Round
SRR2153170	A/A	Round
SRR2153169	A/A	Round
SRR2153168	A/A	Round

SRR2153167	A/A	Round
SRR2153166	A/A	Round
SRR2153165	A/A	Round
SRR2153164	A/A	Round
SRR2153163	A/A	Round
SRR2153162	A/A	Round
SRR2153161	A/A	Round
SRR2153160	A/A	Round
SRR2153159	A/A	Round
SRR2153158	A/A	Round
SRR2153157	A/A	Round
SRR2153156	A/A	Round
SRR2153155	A/A	Round
SRR2153154	A/A	Round
SRR2153153	A/A	Round
SRR2153152	A/A	Round
SRR2153151	A/A	Round
SRR2153150	A/A	Round
SRR2153149	A/A	Round
SRR2153148	A/A	Round
SRR2153147	A/A	Round
SRR2153146	A/A	Round
SRR2153145	A/A	Round
SRR2153144	A/A	Round
SRR2153143	A/A	Round
SRR2153142	A/A	Round
SRR2153141	A/A	Round
SRR2153140	A/A	Round
SRR2153139	A/A	Round
SRR2153138	A/A	Round
SRR2153137	A/A	Round
SRR2153136	A/A	Round
SRR2153135	A/A	Round
SRR2153134	A/A	Round

SRR2153133	A/A	Round
SRR2153132	A/A	Round
SRR2153131	A/A	Round
SRR2153130	A/A	Round
SRR2153129	A/A	Round
SRR2153128	A/A	Round
SRR2153127	A/A	Round
SRR2153126	A/A	Round
Prunus mira	16	NA
SRR3237758	A/A	Round
SRR3237757	A/A	Round
SRR3237756	A/A	Round
SRR3237755	A/A	Round
SRR3237754	A/A	Round
SRR3237752	A/A	Round
SRR3237750	A/A	Round
SRR3237749	A/A	Round
SRR3237748	A/A	Round
SRR3237747	A/A	Round
SRR3237746	A/A	Round
SRR3136174	A/A	Round
SRR3141019	A/A	Round
SRR3136183	A/A	Round
SRR3136181	A/A	Round
SRR3136179	A/A	Round
Prunus ferganensis	5	NA
SRR3138123	A/A	Round
SRR3138121	A/A	Round
SRR3138117	A/A	Round
SRR3138115	A/A	Round
SRR502999	A/A	Round
SRR502998	A/A	Round
Prunus davidiana	5	NA
SRR3237762	A/A	Round

SRR3141018	A/A	Round
SRR3141016	A/A	Round
SRR3138171	A/A	Round
SRR502982	A/A	Round
Prunus avium	1	NA
SRX245854	A/A	Round
Prunus kansuensis	3	NA
SRR3138169	A/A	Round
SRR3138168	A/A	Round
SRR502984	A/A	Round
Prunus serotina	1	NA
SRX272952	A/A	Round
Prunus cerasifera	1	NA
SRX2027264	A/A	Round

Table S7 Primers used in this study

Primer_Name	Primer_Sequence
CAD_F	CTGTTTGTCCATCCTTGCAATTC
CAD_R	AAGTCGATTCTGCTTGCTTCCTT
Chr_6_2_F1	TTGGGCAAATATCTCACATGTCC
Chr_6_2_R1	GGGAACTTCATGGTTGCCATATT
Chr_6_2_F2	TGCCACAAGATGATTAGTGATGC
Chr_6_2_R2	ATTGGTGGGCTCCACCTCTATTA
Chr_6_2_F3	TAACCTGAAAAGCAGGGAAGGAA
Chr_6_2_R3	AACGAAGATTGGTCAAGGGTCAT
Chr_6_2_F4	CACCATTGAAAGTTCGTTTTTGG
Chr_6_2_R4	TGTTGTAAATGGACTTTGGGTGTG
Chr_6_2_F5	ACTGCAGTCGAGAGAGCAAGAGA
Chr_6_2_R5	GTTTGCATTAACCAAGGGCATTT
Chr_6_2_F6	TGTTCCAGAAAACCAAGATGTGC

Chr_6_2_R6	CAATTTTGCGGACAAAGTAGCAG
Chr_6_2_F7	CACACATGACATTGACACGGATT
Chr_6_2_R7	CGAATCTCCTTTCCCGTAGTTTG
